# 急性髓系白血病患者中MLL融合基因筛查及罕见断裂位点病例报道

**DOI:** 10.3760/cma.j.cn121090-20240407-00125

**Published:** 2025-04

**Authors:** 思 李, 焕臣 程, 占影 王, 文鹏 郝, 红 梁, 军 马

**Affiliations:** 哈尔滨市第一医院血液肿瘤研究中心，哈尔滨 150010 Hematology and Tumor Research Center of Harbin First Hospital, Harbin 150010, China

**Keywords:** 基因，MLL, 白血病，髓系，急性, 核型分析, 荧光原位杂交, Gene, MLL, Leukemia, myeloid, acute, Karyotype analysis, Fluorescence in situ hybridization

## Abstract

**目的:**

筛查急性髓系白血病（AML）患者中MLL融合基因阳性患者，分析该融合基因阳性AML患者的临床特征和预后，并报道2例罕见断裂位点病例。

**方法:**

纳入2021年10月至2023年10月哈尔滨市第一医院血液肿瘤研究中心收治的287例AML（非急性早幼粒细胞白血病）患者。男157例，女130例，中位年龄48（19～80）岁。287例患者均进行了白血病相关56种融合基因筛查和染色体核型分析，对融合基因阴性而染色体存在11q23.3易位患者再进行荧光原位杂交（FISH）和转录组测序（RNA-seq）检测。观察指标包括缓解、复发等。

**结果:**

287例AML患者中，MLL融合基因阳性患者15例，阳性率为5.2％。在检测的MLL融合基因的11种类型中，常见融合类型为MLL-ENL（4例）、MLL-ELL（4例）、MLL-AF9（3例）和MLL-AF6（3例）。15例MLL融合基因阳性患者中两例基因检测阴性而染色体和FISH检测阳性，对这两例患者进行RNA-seq检测，1例为MLL融合基因非常见断裂点，1例为新的断裂融合位点。MLL融合基因阳性患者化疗首疗程完全缓解（CR）6例，2个疗程后CR 8例，CR患者中50％ 2个月内出现复发。

**结论:**

RNA-seq技术便于筛查非常规或新断裂位点的融合基因，MLL融合基因阳性患者预后较差。

## Abstract (en)

**Objective:**

To screen for patients with mixed-lineage leukemia（MLL）fusion gene-positive with acute myeloid leukemia（AML）, analyze the clinical characteristics and prognosis of patients with AML with positive fusion genes, and report two cases with rare breakpoint sites.

**Methods:**

This study included 287 patients with AML（non-acute promyelocytic leukemia）admitted to the Hematology and Oncology Research Center of Harbin First Hospital from October 2021 to October 2023. The cohort involved 157 males and 130 females, with a median age of 48 years（range: 19–80 years）. All 287 patients underwent screening for 56 fusion genes and chromosome karyotyping analysis. Fluorescence in situ hybridization（FISH）and RNA seq were further performed on patients with negative fusion genes but positive 11q23.3 chromosomal translocation. Patient observation indicators included remission, recurrence, and others.

**Results:**

Among the 287 patients with AML, 15 were positive for the MLL fusion gene, with a positivity rate of 5.2%. Among the 11 types of MLL fusion genes detected, the most prevalent fusion types were MLL-ENL（4 cases）, MLL-ELL（4 cases）, MLL-AF9（3 cases）, and MLL-AF6（3 cases）. Of the 15 patients with positive MLL fusion gene, 2 demonstrated negative fusion gene while exhibiting positive chromosome and FISH. RNA-seq testing in these two patients revealed an uncommon breakpoint in the MLL fusion gene and a novel breakpoint fusion site, respectively. Complete remission（CR）was achieved in 6 patients with positive MLL fusion genes during the first chemotherapy cycle. After two cycles, eight patients achieved CR, with 50% of them experiencing recurrence within 2 months.

**Conclusion:**

RNA seq technology is useful for screening fusion genes with unconventional or novel cleavage sites, and patients with positive MLL fusion genes demonstrated a poorer prognosis.

MLL（混合谱系白血病，又称KTM2A）基因可调节早期发育和造血过程中的关键基因表达[1]。MLL基因重排可影响造血分化过程导致白血病的发生[2]。5％～10％的初发急性髓系白血病（AML）患者伴MLL基因重排，在治疗相关的AML中约70％的患者存在该基因重排[3]。目前与MLL基因发生融合的“伙伴”基因有八十余种，各亚型的临床异质性较大。如MLL-AF4融合基因大多数见于急性淋巴细胞白血病（ALL），MLL-AF6和MLL-AF9既可见于ALL也可见于AML，而MLL-ELL则主要见于AML患者[4]–[6]。

2016年WHO关于AML更新指南将最常见的t（9;11）归为预后中危组，其余的11q23重排归为预后高危组[7]。MLL融合基因常出现在单核系的AML中，FAB分型以M_4_和M_5_为主[8]。虽然各种MLL融合基因阳性患者临床特征不尽相同，但MLL融合基因阳性和阴性患者相比，大多数恶性程度高，对化疗不敏感，容易出现早期复发且生存期短，总体预后较差[9]–[11]。因此，早期对AML患者进行MLL融合基因筛查十分重要。我们采用荧光定量PCR、染色核型分析和荧光原位杂交（FISH）等方法对MLL重排进行检测，并对其重排类型和特殊病例进行分析，以期为MLL重排阳性患者的预后评估和治疗提供理论依据。

## 病例与方法

1. 病例：标本来自2021年10月至2023年10月哈尔滨市第一医院血液肿瘤研究中心送检的血液病患者骨髓，经形态学初步诊断为AML（急性早幼粒细胞白血病除外）的成人患者287例。男157例，女130例，中位年龄48（19～80）岁。所有患者抽取新鲜骨髓2 ml，送实验室备检。

2. RNA提取及cDNA合成：按照Trizol法提取RNA。逆转录时取1 µg RNA采用白血病相关56种融合基因检测试剂盒所匹配的逆转录试剂盒进行逆转录合成cDNA，逆转录步骤参考试剂盒说明书。

3. 56种融合基因筛查：融合基因筛查采用的是上海源奇生物医药科技有限公司的白血病相关56种融合基因检测试剂盒，该试剂盒中检测的MLL融合基因包括MLL-AF4/AF6/AF9/AF10，MLL-AF17/AFX/SEPT6/AF1q/AF1p和MLL-ENL/ELL，共11种融合类型。按照试剂盒说明书进行荧光定量PCR操作，当内参基因Ct值<30，融合基因Ct值≤33时可判定为该融合基因阳性。

4. 全转录组测序（RNA-seq）分析：样品制备按照上海睿昂基因科技股份有限公司要求进行，依次进行Poly（A）抓取（mRNA富集）、双链cDNA合成、末端修复、接头连接、片段分选、PCR扩增和产物质检等过程，完成文库构建。然后上机测序，测序平台为Illumina HiSeq 2500。

5. 治疗：AML患者诱导化疗采用IA方案（去甲氧柔红霉素+阿糖胞苷），老年或不能耐受强化疗的患者选择阿扎胞苷（AZA）联合维奈克拉。完全缓解（CR）后的巩固治疗主要采用大剂量阿糖胞苷，复发难治的患者采用化疗药物联合靶向药物治疗，复发后的挽救治疗采用CLAG方案（克拉屈滨+阿糖胞苷+G-CSF）。

6. 随访：所有入组患者均自确诊之日开始随访，随访资料来源于住院病历、门诊病历及电话随访记录，截止时间为2023年12月31日。对于随访期间的死亡病例按照病历记录或与患者家属电话联系确定死亡日。

## 结果

1. MLL融合基因阳性AML患者基本情况：在白血病相关56种融合基因筛查和染色体核型分析的287例AML患者中，MLL融合基因阳性患者15例，阳性率为5.2％。在MLL融合基因阳性患者中MLL-ENL和MLL-ELL阳性各4例，占26.7％；MLL-AF6和MLL-AF9阳性各3例，占20.0％；MLL-AF1q仅1例阳性，其余六种融合类型未检出。15例阳性患者的FAB分型：M_5_ 6例，M_2_ 2例，其余7例为未分型AML。15例患者中首疗程诱导化疗后CR 6例，2个疗程后CR 8例，7例患者2个疗程未缓解，缓解的患者中4例2个月内复发。本文列举的2例特殊断裂位点的AML复发患者在观察期均死亡，1例患者总生存期4个月，另1例患者总生存期6个月。

2. 染色体、FISH和融合基因综合分析：综合分析这15例MLL融合基因阳性患者，其中有12例患者染色体结果显示存在11q23.3易位，3例患者为复杂核型，3例患者为正常核型，详见表1。两例患者染色体核型分析存在11号染色体易位而融合基因检测为阴性，为了进一步分析患者MLL异常情况，加做了MLL断裂探针的FISH检测，结果表明这两例患者FISH检测的MLL断裂探针结果均为阳性，和染色体结果相符，对这2例患者进行RNA-seq和一代测序分析，表明这2例患者中1例为非常见的断裂位点，1例为新的融合位点，试剂盒中均未包含该片段的引物和探针。

**表1:** 15例MLL融合基因阳性急性髓系白血病患者临床特征

例号	性别	年龄（岁）	融合基因	染色体	突变基因
1	男	80	MLL-ENL^a^	49，XY, +8, +20, +21, t（11;19）（q23.3; p13.3）	RUNX1、SRSF2
2	女	29	MLL-ELL^a^	47，XX, +13, t（11;19）（q23.3; p13.1）	FLT3-ITD、PTPN11、WT1
3	男	63	MLL-AF1Q	46，XY, t（1;11）（q21; q23）, +8, −5, del（20）（q11.2q13.3）	ASXL1、IDH1、SRSF2
4	男	78	MLL-AF6	46，XY, t（6;11）（q27; q23.3）	TET2、TP53
5	女	24	MLL-AF9	46，XX	WT1
6	女	43	MLL-ELL	46，XX, t（11;19）（q23.3; p23.1）	NRAS
7	女	42	MLL-AF9	46，XX, t（9;11）（p21.3; q23.3）	阴性
8	女	51	MLL-AF9	46，XX, t（9;11）（p21.3; q23.3）	BCOR、FLT3-ITD
9	男	45	MLL-ENL	46，XY	KRAS、NOTCH1
10	男	48	MLL-ELL	46，XY	FLT3-ITD
11	女	42	MLL-ENL	46，XX, t（11;19）（q23.3; p13.3）	阴性
12	男	19	MLL-ELL	46，XY, t（11;19）（q23.3; p13.1）	PHF6、WT1、BRAF
13	女	62	MLL-ENL	46，XX, +8, t（11;19）（q23.3; p13.3）	阴性
14	女	71	MLL-AF6	44，XX, −3, +8, −12, −18, t（6;11）（q27; q23.3）	NRAS
15	女	52	MLL-AF6	46，XX, t（6;11）（q27; q23.3）	BCOR、NRAS

**注** ^a^最初白血病相关56种融合基因筛查阴性而染色体核型结果阳性，后来采用RNA-seq方法检测，又根据断裂位点重新设计引物后重新筛查出的融合基因阳性

3. MLL融合基因阳性患者突变分析：分析15例MLL融合基因阳性患者二代测序结果表明，12例患者存在AML相关基因突变，突变阳性率为80.0％，阳性患者中突变基因中位个数为2（1～3）个。其中RAS信号通路（NRAS、KRAS和PTPN11）基因突变率最高，该类突变阳性患者占比33.3％（5/15），其次为FLT3-ITD突变和WT1突变，突变阳性患者占比均为20.0％（3/15），再次为SRSF2基因，15例患者中有2例SRSF2突变，且突变阳性的2例患者均为复杂核型。其余均为单个基因的非重复性突变，具体结果见表1。

4. 2例罕见断裂位点患者病例资料：

例1，男，80岁。因无明显诱因出现下颌肿痛伴发热就诊，骨髓细胞形态学结果显示患者为AML-M_5_，原始单核细胞比例为62％；流式细胞术检测结果显示87.47％细胞为异常的髓系幼稚细胞，不除外AML；二代测序结果显示该患者存在RUNX1和SRSF2突变；染色体结果为49,XY, +8, +20, +21, t（11;19）（q23.3; p13.3）；FISH结果显示MLL-ENL阳性，阳性率72％；但白血病相关56种融合基因筛查MLL融合基因阴性，且MLL-ENL融合基因定量结果也为阴性。为了进一步验证，将该患者标本进行RNA-seq检测，结果显示MLL-ENL融合基因阳性，断裂融合位点为11:118355029-19:6230724（图1），为非常见断裂融合位点。根据该结果重新设计MLL-ENL融合基因定量引物，然后重新进行定量分析后MLL-ENL定量结果为18.58％。同时又将该引物加入到56种融合基因筛查中，然后重新定性筛查后MLL-ENL也出现阳性结果。采用SAV方案（塞利尼索、阿扎胞苷和维奈克拉）联合化疗1个疗程后缓解，染色体变为正常核型，融合基因结果转为阴性，但1个月后复发，MLL-ENL融合基因表达显著性升高，染色体又变为复杂核型，化疗未缓解，最后患者伴发肺感染死亡。

**图1:**
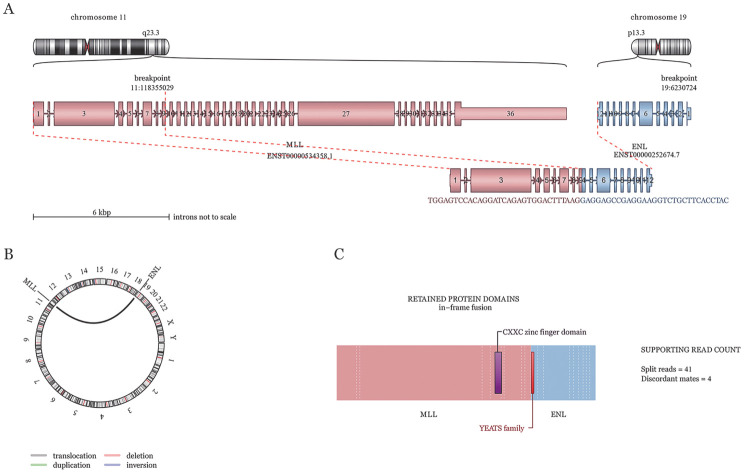
RNA-seq检测例1 MLL-ENL断裂融合 **A** MLL::ENL融合基因结构示意图，MLL::ENL的断点定位于染色体11q23.3和染色体19p13.3；**B** MLL::ENL染色体易位圈图；**C** MLL::ENL融合基因形成所保留的蛋白质结构域

例2，女，29岁。骨髓细胞形态学结果显示患者为AML-M_5_，原始幼稚单核比例为84％；流式细胞术检测显示幼稚单核细胞占有核细胞的21.81％，考虑为AML；二代测序结果显示该患者存在FLT3、PTPN11和WT1突变，染色体结果为47，XX, +13, t（11;19）（q23.3; p13.1），而白血病相关56种融合基因筛查全阴性，加做MLL的断裂探针FISH，结果表明MLL断裂阳性率为80％，为了查明融合基因筛查阴性的原因，我们做了RNA-seq检测，结果表明该患者存在MLL-ELL的exon10-exon8融合，断裂融合位点为11:118357813-19:18561849（图2）。根据该融合类型重新设计引物后进行荧光定量PCR，并把56种融合基因筛查中也加入了该引物，重新检测后定量和筛查结果均表明该患者为MLL-ELL阳性。患者也采用SAV方案化疗1个疗程后形态学缓解，但流式细胞术检测显示微小残留病（MRD）仍为阳性，化疗3个疗程后缓解，缓解两个月后复发，后采用CLAG方案联合脂质体米托蒽醌治疗未缓解，后又出现脑出血，患者放弃治疗死亡。

**图2:**
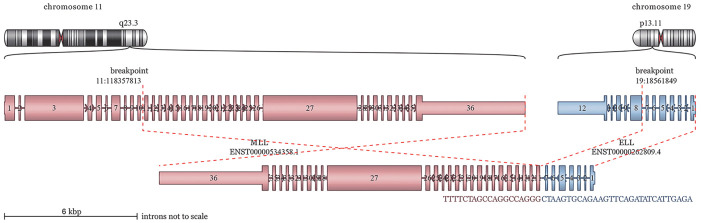
RNA-seq检测例2 MLL::ELL断裂融合融合基因结构示意图：MLL::ELL的断点定位于染色体11q23.3和染色体19p13.11（MLL::ELL融合基因由于反义转录，没有保留蛋白结构域)

## 讨论

2016年WHO指南将AML伴t（9;11）（p21.3;q23.3）和MLLTT3-MLL列为AML的单独类型，2022年WHO指南进行更新将AML伴MLL重排列为单独类型。伴MLL重排的AML成人患者具有高白细胞、单核细胞分化倾向等的临床特征，且均与预后不良相关。本组15例MLL融合基因阳性患者中7例患者FAB分型为M_5_，2个疗程化疗后7例未缓解，缓解患者中有4例两个月内复发，与文献报道的单核细胞分化倾向和预后不良吻合[7],[12]–[13]。

本研究15例MLL融合基因阳性患者中有12例出现基因突变，突变阳性率达80.0％，表明MLL融合基因阳性患者易伴随基因突变，与Lavallée等[14]的研究结果类似。不良基因突变的伴随出现加剧患者的预后不良，这也可能是MLL基因重排患者预后不良的一个原因。突变阳性患者中约33.3％的患者出现了RAS信号通路相关基因突变，其次为FLT3-TID突变，与Lavallée等[14]的研究结果相似。2例SRSF2突变阳性患者均为复杂核型，表明剪接因子基因SRSF2易伴随复杂核型出现，同时也说明该基因突变可能提示预后不良，与2023年WHO指南提出的SRSF2基因突变为独立预后不良指标比较吻合[15]。

MLL基因重排在2022年WHO分类中被确认为一个独特的亚型，预后和治疗有其独特性。因此，MLL融合基因筛查对确诊该亚型AML进而对患者进行个体化治疗至关重要。目前我们检测融合基因的方法主要有染色体核型分析、FISH、荧光定量PCR和RNA-seq。染色体核型虽然可一次可分析所有染色体异常，但其敏感性较低（约5％），对于一些细微的缺失（5 Mb以内）或重复难以辨别。FISH可检测片段长度在400 kb～5 Mb，其可检测一些染色体检测不到的小片段异常，敏感性为0.2％～1％，但该方法受探针的限制，只能检测特定的基因异常[16]–[17]。相比于以上两种检测方法，荧光定量PCR敏感性最高，为0.01％～0.001％，且后续可进行MRD监测，但其检测片段较短，一般在200 bp以内，并且检测片段受引物限制，只能检测常见的断裂融合位点[18]。RNA-seq是近年来发展起来的高通量测序技术，它可以覆盖所有断裂位点，但其敏感性仅为10％[19]，且检测周期长、价格较高。因此，我们要选择合适的检测方法，必要时几种检测方法综合运用来提高MLL融合基因阳性检出率，使更多患者能获得个体化诊疗。本文中的两例特殊断裂位点病例均是这样筛查出来的，尤其是第2例患者。我们查阅相关文献发现目前已确定的MLL断裂点主要分布在MLL的exon7～14，ELL断裂点主要集中在exon2和exon3，罕见型MLL-ELL融合类型为exon10-exon3、exon9-exon6、exon20-exon2和exon6-exon2等[20]–[21]，未检索到该患者MLL-ELL（exon10-exon8）的融合类型，该患者为新的MLL-ELL融合类型，患者化疗3个疗程后缓解，2个月后复发，复发后再次化疗未缓解，随后发生脑出血死亡，患者总生存期不到6个月，说明了这一融合基因类型与其他MLL融合基因类型一样，也是预后不良甚至更差，但这一新的融合基因类型预后是否与其他融合基因类型存在区别，还需要后续大量阳性患者结果的验证。
